# Magnetic Nanoconjugated Teicoplanin: A Novel Tool for Bacterial Infection Site Targeting

**DOI:** 10.3389/fmicb.2018.02270

**Published:** 2018-10-17

**Authors:** Ilaria Armenia, Giorgia Letizia Marcone, Francesca Berini, Viviana Teresa Orlandi, Cristina Pirrone, Eleonora Martegani, Rosalba Gornati, Giovanni Bernardini, Flavia Marinelli

**Affiliations:** Department of Biotechnology and Life Sciences, University of Insubria, Varese, Italy

**Keywords:** antibiotic resistance, iron oxide nanoparticles, glycopeptide antibiotics, antimicrobial activity, teicoplanin, *Staphylococcus aureus* biofilm

## Abstract

Nanoconjugated antibiotics can be regarded as next-generation drugs as they possess remarkable potential to overcome multidrug resistance in pathogenic bacteria. Iron oxide nanoparticles (IONPs) have been extensively used in the biomedical field because of their biocompatibility and magnetic properties. More recently, IONPs have been investigated as potential nanocarriers for antibiotics to be magnetically directed to/recovered from infection sites. Here, we conjugated the “last-resort” glycopeptide antibiotic teicoplanin to IONPs after surface functionalization with (3-aminopropyl) triethoxysilane (APTES). Classical microbiological methods and fluorescence and electron microscopy analysis were used to compare antimicrobial activity and surface interactions of naked IONPs, amino-functionalized NPs (NP-APTES), and nanoconjugated teicoplanin (NP-TEICO) with non-conjugated teicoplanin. As bacterial models, differently resistant strains of three Gram-positive bacteria (*Staphylococcus aureus*, *Enterococcus faecalis*, and *Bacillus subtilis*) and a Gram-negative representative (*Escherichia coli*) were used. The results indicated that teicoplanin conjugation conferred a valuable and prolonged antimicrobial activity to IONPs toward Gram-positive bacteria. No antimicrobial activity was detected using NP-TEICO toward the Gram-negative *E. coli.* Although IONPs and NP-APTES showed only insignificant antimicrobial activity in comparison to NP-TEICO, our data indicate that they might establish diverse interaction patterns at bacterial surfaces. Sensitivity of bacteria to NPs varied according to the surface provided by the bacteria and it was species specific. In addition, conjugation of teicoplanin improved the cytocompatibility of IONPs toward two human cell lines. Finally, NP-TEICO inhibited the formation of *S. aureus* biofilm, conserving the activity of non-conjugated teicoplanin versus planktonic cells and improving it toward adherent cells.

## Introduction

According to a recent survey of the World Health Organization (WHO, 2017), antibiotic resistance represents one of the greatest threats to global health today and contributes significantly to longer hospital permanence, higher medical costs, and increased mortality. At least 700,000 people die annually because of infections caused by resistant bacteria. This number is predicted to increase upto 10 million by 2050 and is consequentially associated with a social and economic burden. This public health threat is exacerbated by the paucity of novel antibiotics expected to enter clinical use in the near future (Fedorenko et al., 2015). A corollary to acute illness is the increased number of chronic bacterial infections due to the prevalence of biofilm colonization (Arciola et al., 2018). Currently, medical device-related infections account for more than 60% of all the hospital-acquired infections in the United States (Weiner et al., 2016). Biofilms are complex, three-dimensional bacterial communities living in a self-produced extracellular matrix. The biofilm-forming bacteria survive better than their free-living (planktonic) counterparts in hostile environments; they are 10 to 100 times less susceptible to antimicrobial agents and are protected against the host immune system, making the treatment of these infections quite challenging (Davis, 2003; Venkatesan et al., 2015).

One promising approach in the field of antimicrobial therapy is the use of nanotechnology-tailored agents for preventing and treating infections caused by resistant bacteria. Unique and well-defined features distinguish nanoparticles (NPs) from their bulk counterparts, such as large surface area-to-volume ratio and dimensions that are comparable to those of biomolecules, effectively providing a platform with a high number of functional sites and possible interactions with bacterial cells and biofilms. Of all the NPs tested for antimicrobial activity thus far, silver NPs (AgNPs) have been studied most intensively (Natan and Banin, 2017). Although researchers have widely agreed that the broad-spectrum antibacterial activity of AgNPs can be predominantly ascribed to the release of Ag ions, AgNPs demonstrate unique properties because they adhere to the bacterial surface, altering membrane properties and thus delivering Ag ions more effectively to the bacterial cytoplasm and membrane (Durán et al., 2016). Consequently, the antibacterial effect of AgNPs is observed at concentrations with a 10-fold lower magnitude than those used for bulk Ag ions. The antibacterial activity of AgNPs is reported to be mediated by a multiplicity of still-not-completely understood mechanisms following their interaction with the bacterial surface, which act in parallel (i.e., oxidative stress, membrane depolarization, and protein and DNA interaction), thus explaining why bacterial resistance does not easily arise (Hajipour et al., 2012; Natan and Banin, 2017; Baranwal et al., 2018). Very recent studies (Xiang et al., 2017; Xie et al., 2017, 2018) show that the antibacterial activity of AgNPs may be successfully exploited in preparing nanocomposite materials to be used as antibacterial coatings of titanium-based metallic implants and poly(ether ether ketone) medical devices, which are both widely employed in dentistry and orthopedic applications. Entrapping AgNPs in graphene oxide nanosheets wrapped with a thin layer of collagen (Xie et al., 2017), in hybrid polydopamine/graphene oxide coatings (Xie et al., 2018), or in biocompatible polymers such as poly(lactic-*co*-glycolic) acid (Xiang et al., 2017) endows medical implants with a long-lasting self-antibacterial activity. *In vivo* studies using these innovative coatings in animal models confirm that combining the unique properties of different nanomaterials prevents bacterial infection and provides a good cytocompatibility of the medical devices (Xie et al., 2017, 2018).

A synergic, but as yet less exploited strategy when developing nano-based antimicrobial agents involves using NPs as nanocarriers for antibiotics, taking advantage of the high surface-to-volume ratio platform that they offer for attaching a large number of molecules. The advantages of using NPs in this way depend on the nature of both the NPs and the drugs under consideration, as recently reviewed (Natan and Banin, 2017). These advantages might include (i) protecting the nanoconjugated drug from degradation and oxidation; (ii) increasing drug solubility, antimicrobial activity, and biodistribution; (iii) delivering the antibiotic to the site of the infection; and (iv) enhancing drug penetration into biofilms, facilitating the killing of encased bacteria. As antibiotic nanocarriers, iron oxide nanoparticles (IONPs) have recently attracted increased interest thanks to their unique magnetic properties (Dinali et al., 2017). In fact, IONPs can be guided by an external magnetic field to a targeted organ/biofilm and specifically localized at the site of infection (Wu et al., 2015; Stepien et al., 2018). In addition, IONPs are easily produced and functionalized, and they possess a high drug-loading capacity, low cell toxicity, and high biocompatibility (Ali et al., 2016; Dinali et al., 2017). In the last decade, relatively few studies have investigated the potential of surface-modified IONPs as antibacterial agents in depth. Core-shell Fe_3_O_4_-AgNPs were tested as antimicrobial agents against Gram-positive and Gram-negative bacteria where the silver shell was responsible for antimicrobial action (Chudasama et al., 2009). Biocompatible polyvinyl alcohol-coated IONPs were used in biomedical applications and reported to be active against *Staphylococcus aureus* in a dose-dependent manner (Tran et al., 2010). Similarly, chitosan-coated IONPs were shown to have a higher antimicrobial activity than naked IONPs due to the positive surface potential, which interacted better with negatively charged bacterial cell surfaces (Arakha et al., 2015a). According to other authors (Huang et al., 2010; Ebrahiminezhad et al., 2014), IONP surface functionalization with (3-aminopropyl) triethoxysilane (APTES) elicited an antimicrobial effect by creating a high density of amino groups, which could interact with negatively charged sites on the bacterial cells through electrostatic interactions. The well-developed surface chemistry of IONPs made it possible to incorporate a variety of commonly used antibiotics such as the β-lactam amoxicillin, penicillin, and ampicillin, the aminoglycoside streptomycin, and the glycopeptide vancomycin (Chifiriuc et al., 2013; Grumezescu et al., 2014; Hussein-Al-Ali et al., 2014; El Zowalaty et al., 2015; Wang et al., 2017), providing evidence that biocompatible magnetic NPs might enable site-specific antibiotic delivery. Vancomycin-carrying, folic acid-tagged chitosan NPs were successfully used to deliver vancomycin to bacterial cells (Chakraborty et al., 2010, 2012), and vancomycin-modified mesoporous silica NPs were used for selective recognition and killing of Gram-positive bacteria over macrophage-like cells (Qi et al., 2013). An alternative use of IONPs functionalized with vancomycin – an antibiotic that binds to bacterial cell walls – was to apply them as ligands for the affinity capture of a wide range of bacteria from biological samples, including Gram-positive bacteria such as *S. aureus* and Gram-negative bacteria such as *Escherichia coli* (Gu et al., 2003; Lin et al., 2005; Kell et al., 2008). Because of the magnetic properties of vancomycin-functionalized IONPs, vancomycin-captured bacteria can be magnetically separated and concentrated from large volumes into much smaller volumes, allowing bacterial analysis and detection based on, for example, genomic DNA (Kell et al., 2008; Zhu et al., 2015).

In this work, we employed IONPs as carriers of the lipoglycopeptide antibiotic teicoplanin, which has been used in clinical practice since 1988 in Europe and 1998 in Japan. Teicoplanin is considered a drug of “last resort” for treating severe infections by multiresistant Gram-positive pathogens, including the methicillin-resistant *S. aureus* (MRSA) and the anaerobe *Clostridioides difficile* (Marcone et al., 2018). Teicoplanin is a complex molecule with a peptide core of seven aromatic amino acids tailored with sugar residues, chlorine atoms, methyl groups, and a lipid chain. It forms five specific hydrogen bonds with the D-alanyl-D-alanine terminus of the peptidoglycan precursors of the bacterial cell wall, blocking its synthesis and consequently causing cell lysis (Binda et al., 2014). The antibacterial spectrum of teicoplanin activity against Gram-positive bacteria is similar to that of vancomycin, but teicoplanin shows an increased potency, particularly against some resistant clinical isolates belonging to *Staphylococcus*, *Streptococcus*, and *Enterococcus* genera (Van Bambeke, 2006). In addition, teicoplanin is active on vancomycin-resistant enterococci with VanB-phenotype (Van Bambeke, 2006; Binda et al., 2014). The superior antimicrobial potency of the lipoglycopeptide teicoplanin in comparison to the glycopeptide vancomycin is due to the *in vivo* membrane anchoring of the hydrophobic tail of teicoplanin, which strengthens the bond to membrane-localized peptidoglycan precursors and promotes synergic back-to-back dimerization of antibiotic molecules (Allen and Nicas, 2003; Treviño et al., 2014). In addition, lipidation seems to represent the key functional difference between vancomycin and teicoplanin, which is related to their differing abilities of inducing glycopeptide antibiotic resistance response in enterococci and actinomycetes (Dong et al., 2002; Binda et al., 2018). To the best of our knowledge, this is the first study exploring the feasibility of conjugating teicoplanin to IONPs and testing the potential of nanoconjugated teicoplanin as a promising tool for treating bacterial infections caused by resistant bacteria.

## Materials and Methods

### Materials

All chemical reagents, including acetonitrile (CH_3_CN), ammonium formate (HCOONH_4_), ammonium hydroxide (NH_4_OH), APTES, boric acid (H_3_BO_3_), crystal violet (C_25_N_3_H_30_Cl), 2′,7′-dichlorodihydrofluorescein (DCFH-DA), *N*-(3-dimethylaminopropyl)-*N*′-ethylcarbodiimide hydrochloride (EDC), ethanol (C_2_H_6_O), ferric nitrate [Fe(NO_3_)_3_ × 9H_2_O], formaldehyde (CH_2_O), glutaraldehyde (C_5_H_8_O_2_), iron dichloride (FeCl_2_ × 4H_2_O), iron trichloride (FeCl_3_ × 6H_2_O), *N*-hydroxysuccinimide (NHS), nitric acid (HNO_3_), osmium tetroxide (OsO_4_), phosphate-buffered saline (PBS), sodium cacodylate (C_2_H_7_AsO_2_), sodium chloride (NaCl), sodium hydroxide (NaOH), sodium 2-(*N*-morpholino)ethanesulfonic acid hemisodium salt (MES), and teicoplanin, were purchased from Sigma-Aldrich, Milan, Italy. The LIVE/DEAD BacLight fluorescence assay kit was purchased by Thermo Fisher Scientific, Monza, Italy. Epon-Araldite 812 was purchased from Electron Microscopy Sciences, Hatfield, PA, United States. All the chemical reagents were used without additional purification.

### Microbial Strains and Culture Conditions

*Escherichia coli* ATCC 35218, *Bacillus subtilis* ATCC 6633, *S. aureus* ATCC 6538P (methicillin susceptible *S. aureus*, MSSA), *S. aureus* ATCC 43300 (MRSA), *Enterococcus faecalis* ATCC 29212, and *E. faecalis* ATCC 51299 (VanB phenotype) were obtained from the American Type Culture Collection (ATCC). *E. faecalis* 9160188401-EF-34 (VanA phenotype) is a clinical isolate, which was kindly provided by Laboratorio Microbiologia Clinica – Ospedale di Circolo, Varese, Italy. *E. coli* and *B. subtilis* were propagated overnight in Luria Bertani medium (LB, 2% tryptone, 2% yeast extract, and 1% NaCl), and the *S. aureus* and *E. faecalis* strains in Müller Hinton broth 2 (MHB2, 0.3% beef infusion solids, 1.75% casein hydrolysate, and 0.15% starch) with continuous shaking at 200 rpm and 37°C. For exponential growth, overnight cultures were transferred to fresh medium: inocula were prepared to start the cultures with an optical density at 600 nm (OD_600 nm_) of 0.1 in the final medium. For long-term preservation, bacterial cultures were stored at -20°C in 20% glycerol. Media were acquired from Sigma-Aldrich, Milan, Italy, unless otherwise stated.

### Synthesis of the IONPs

Iron oxide (Fe_2_O_3_) NPs were synthesized using the coprecipitation method reported by Balzaretti et al. (2017). Briefly, under vigorous stirring for 30 min, 8.89 g of FeCl_3_ × 6H_2_O and 3.28 g FeCl_2_ × 4H_2_O were mixed in 380 mL of water, while slowly adding 1.5 mL of HCl (37%) dropwise into the solution to completely dissolve the salts. Following this step, 25 mL of NH_4_OH (25%) was added. Particles were washed several times with Milli-Q water and 40 mL of 2 M HNO_3_ was added and heated at 90°C for 5 min. Then, particles were separated by a magnet from the reaction mixture; subsequently, 60 mL of 0.34 M solution of Fe(NO_3_)_3_ × 9H_2_O was added. The suspension was heated at 90°C for 30 min. The supernatant was removed and IONPs were collected by a magnet, suspended in Milli-Q water, and left in dialysis overnight. IONPs were stored at 4°C.

### Functionalization With APTES

To prepare functionalized IONPs, a standard protocol (Balzaretti et al., 2017) was followed: a 1.5 M solution of APTES in ethanol was added to 150 mg of IONPs and stirred for 1 h at room temperature. Then, the temperature was increased to 90°C and the solution was stirred for an additional hour. The amino-modified IONPs (NP-APTES) were collected by a magnet, washed several times, and suspended in Milli-Q water.

### Teicoplanin Conjugation to NP-APTES

To prepare teicoplanin-conjugated NPs (NP-TEICO), a solution containing teicoplanin (500 μg), 13 mM EDC, and 26 mM NHS was prepared and added to the NP-APTES (4 mg/mL) dispersed in 30 mM MES buffer at pH 6.0 in a final volume of 1 mL. The reaction was mixed for 6 h at room temperature. NP-TEICO were washed twice and resuspended in fresh 30 mM MES buffer at pH 6.0.

### Characterization of NPs

The shape, size, and size distribution of IONPs, NP-APTES, and NP-TEICO were investigated by transmission electron microscopy (TEM) using a JEOL 1010 electron microscope (Tokyo, Japan). Samples for TEM were dispersed in Milli-Q water on carbon-coated copper grids and dried at room temperature. The hydrodynamic diameter size and polydispersity index (PDI) of IONPs, NP-APTES, and NP-TEICO were measured in 0.9% NaCl. Zeta potential was measured on samples diluted in 1 mM KCl at 25°C. Measurements were performed at 25°C using a 90 Plus Particle Size Analyzer (Brookhaven Instruments Corporation, NY, United States).

### HPLC Analysis

Teicoplanin was measured by HPLC according to the method previously reported in Taurino et al. (2011). HPLC analyses were performed on a 5-μm particle size Symmetry C18 (VWR International LCC, Radnor, PA, United States) column (4.6 mm × 250 mm). The column was eluted at a 1 mL/min flow rate with a 30-min linear gradient from 15 to 65% of Phase B, followed by 10 min with 100% Phase B. For Phase A we used a 32 mM HCOONH_4_, pH 7.0:CH_3_CN 90:10 (v/v) mixture, and for Phase B a 32 mM HCOONH_4_, pH 7.0:CH_3_CN 30:70 (v/v) mixture. Chromatography was performed with a model 1100 HPLC system (Elite Lachrom Hitachi LLC, VWR, Milan, Italy) and UV detection was at 236 nm.

### Agar Diffusion Test

Antimicrobial activities of IONPs, NP-APTES, and NP-TEICO were tested against *E. coli* ATCC 35218, *B. subtilis* ATCC 6633, and *S. aureus* ATCC 6538P by employing an agar diffusion assay (Finn, 1959). Briefly, bacterial cultures were grown in MHB2 until an OD_600 nm_ of 0.4 was reached and then used to prepare agar plates containing Müller-Hinton Agar (MHA). 10 μL of IONPs, NP-APTES, NP-TEICO (4 mg/mL loaded with 500 μg/mL of teicoplanin in the case of NP-TEICO), and of teicoplanin (500 μg/mL) in 30 mM MES buffer, pH 6.0, were loaded manually onto the inoculated plates. The plates were incubated at 37°C for 24 h. The diameters of the zones of bacterial growth inhibition surrounding the droplets were measured.

### Determination of Minimum Inhibitory Concentration and Minimum Bactericidal Concentration

Minimum inhibitory concentrations (MICs) of non-conjugated and nanoconjugated teicoplanin were determined toward *B. subtilis*, *S. aureus*, and *E. faecalis* strains by applying the broth dilution method using MHB2, as recommended by the Clinical and Laboratory Standards Institute guidelines (CLSI, 2018). About 5 × 10^5^ exponentially growing bacterial cells were inoculated into MHB2 containing increasing concentrations of teicoplanin and NP-TEICO in 30 mM MES buffer, pH 6.0, and shaken for 16–20 h at 37°C. NP-TEICO concentrations to be added were calculated considering the amount of teicoplanin loaded onto IONPs (nanoconjugated teicoplanin) under the reaction conditions described above. MICs were the minimal concentrations of nanoconjugated and non-conjugated teicoplanin at which no turbidity could be detected.

To evaluate the minimum bactericidal concentrations (MBCs), 100 μL of bacterial cultures used for the MIC test were plated onto MHA and incubated at 37°C for 24 h. MBCs were the minimal concentrations of nanoconjugated and non-conjugated teicoplanin at which no growth could be detected. The tolerance level of each tested bacterial strain toward nanoconjugated and non-conjugated teicoplanin was determined according to May et al. (1998) using the following formula: Tolerance = MBC/MIC.

### Growth Kinetic Analysis

Growth kinetics of *B. subtilis* ATCC 6633, *S. aureus* ATCC 6538P, and *E. coli* ATCC 35218 populations were followed by measuring OD_600 nm_ using an UV-Vis V-560 Spectrophotometer (JASCO, MD, United States) at regular time intervals. Preinocula were prepared from overnight cultures in LB or MHB2 at 37°C and at 200 rpm. Experiments were conducted in 50-mL tubes containing a final volume of 10 mL of LB or MHB2 added after 1 h of growth from inocula with equivalent volumes of IONPs, NP-APTES, and NP-TEICO preparations (4 mg/mL) previously resuspended in 30 mM MES buffer, pH 6.0, or with the teicoplanin control solution (500 μg/mL).

### Viability Assay

Viable counts (expressed as colony-forming units per mL, CFU/mL) were estimated by employing the plate count technique. For CFU measurement, a standard volume (10 μL) of undiluted or serially diluted samples collected from stationary phase cultures on treatment with teicoplanin, IONPs, NP-APTES, and NP-TEICO, as reported above, were plated on nutrient agar. Plates were incubated for 24 h at 37°C to evaluate the viable cells.

### Fluorescence Microscopy Analysis

To investigate the effect of IONPs, NP-APTES, and NP-TEICO on bacterial cells, the LIVE/DEAD BacLight fluorescence assay was used (L7007, Molecular probes, Thermo Fisher Scientific). Following the manufacturer’s protocol, bacteria were cultivated overnight at 37°C and agitated at 200 rpm, appropriately diluted, and treated for 5 h with 4 mg/mL of IONPs, NP-APTES, and NP-TEICO and teicoplanin (500 μg/mL). From these cultures, 10 mL of each bacterial solution was centrifuged at 7000 rpm for 15 min. The supernatants were discarded and the pellets were suspended in saline solution (0.9%). The samples were incubated at room temperature for 1 h (mixing every 15 min) and then washed twice with saline solution. Finally, the pellets were resuspended in an equal volume of saline solution (0.9%). Then, 3 μL of dye mixture was added to each 1 mL of the prepared bacterial samples and incubated in the dark for 15 min after properly mixing the bacterial suspensions. Fluorescence images were taken by trapping 5 μL of stained bacterial samples between a slide and a cover slip. For imaging the samples, an optical microscope with appropriate filters was employed (Axiophot; Carl Zeiss, Milan, Italy). ImageJ (Schneider et al., 2012) was used to quantify total fluorescence intensity of the bacteria. Intensities were expressed as percentage (%) relative to the saturation fluorescence within the field; red and green fluorescence stains corresponded to live or dead bacteria, respectively (Borcherding et al., 2014; Arakha et al., 2015a,b).

### Transmission Electron Microscopy Analysis

The interaction pattern of NPs with bacteria was also studied by TEM. After 5 h of exposure to 4 mg/mL IONPs, NP-APTES, NP-TEICO, or teicoplanin (500 μg/mL), pellets were washed with PBS and fixed in Karnovsky solution (4% formaldehyde, 2% glutaraldehyde, 0.1 M sodium cacodylate, pH 7.2) overnight at 4°C. The samples were washed three times with 0.1 M sodium cacodylate for 10 min and postfixed in the dark for 1 h with 1% osmium tetroxide in 0.1 M sodium cacodylate buffer, pH 7.2, at room temperature. After dehydration with a series of ethyl alcohol, the samples were embedded in an Epon-Araldite 812 1:1 mixture. Thin sections (90 nm), obtained with a Pabisch Top-Ultra ultramicrotome (Emme 3 S.r.l., Milan, Italy), were observed with a Morgagni electron microscope (Philips, Eindhoven, Netherlands) operated at 80 keV.

### Biofilm Assay

*S. aureus* ATCC 6538P cultures, grown overnight in LB, were diluted in fresh medium to reach a cell density of 10^7^ CFU/mL and dispensed in 24-well plates, adding increasing concentrations of nanoconjugated or non-conjugated teicoplanin (2.5, 5, and 10 μg/mL) and of naked IONPs or NP-APTES (20, 40, and 80 μg/mL). The amounts of NPs to be added took into account the teicoplanin loaded on NPs under the reaction conditions described above. Following incubation at 37°C for 24 h, the adherent biomass was quantified by crystal violet (CV) staining. Biofilms were stained with CV 0.1% for 20 min, washed twice with PBS, and air dried overnight at room temperature; the CV was then dissolved in 33% acetic acid for 10 min. The amount of solubilized dye was spectrophotometrically measured at 595 nm (Infinite 200 PRO; TECAN, Männedorf, Switzerland). To assess the effect of teicoplanin and of NP preparations on the cell viability of planktonic and adherent cell subpopulations, cells from the planktonic phase were collected and adherent cells were recovered by scraping the wells and then suspended in 1 mL of phosphate buffer. Cultures were diluted and CFU were estimated by plate counting in MHA plates. Viable counts of planktonic cells were expressed as CFU/mL and adherent cells as CFU per well (CFU/well).

To test the effect of teicoplanin and NP preparations on biofilm dispersal, biofilms were prepared as indicated previously and incubated at 37°C for 48 h before adding nanoconjugated or non-conjugated teicoplanin (5, 25, and 50 μg/mL) and naked IONPs or NP-APTES (40, 200, and 400 μg/mL). Following 24-h incubation at 37°C, biofilm biomass was evaluated by CV staining and the cell viability of adherent and planktonic cells was estimated by applying the viable count technique, as previously described.

### Cell Cultures

Two different cell lines were used to evaluate NP-TEICO *in vitro* cytotoxicity: a tumor model SKOV-3 cell line from ovarian adenocarcinoma and a non-tumor cell line, hASCs (human adipose-derived stem cells). SKOV-3 cells were cultured as reported in the literature (Cappellini et al., 2015). hASCs were isolated and cultured as previously reported (Palombella et al., 2017).

### Cytotoxicity Test

Cell cytotoxicity was determined by measuring ATP content using the RealTime-Glo^TM^ MT Cell Viability Assay (Promega, Milan, Italy) according to the manufacturer’s instructions. Briefly, 500 cells were plated in 96-well plates in 200 μL of cell medium (RPMI for SKOV-3 and DMEM/DMEM F12 1:1 for hASC). After 24 h, the cells were exposed to increasing concentrations of nanoconjugated or non-conjugated teicoplanin or to the corresponding concentrations of NPs (considering the teicoplanin loaded per mg of NPs) and then a solution 2× the substrate and NanoLuc^®^ Enzyme were added. The cells were incubated at 37°C and in 5% CO_2_-humidified atmosphere, and luminescence was read every 24 h using the Infinite F200 plate reader (Tecan Group, Männedorf, Switzerland).

### Statistics

All experiments were repeated at least three times on separate dates. Mean and standard deviation (SD) calculations were performed using Microsoft Excel 2003 (Microsoft Corporation, Redmond, WA, United States). Data were analyzed by means of one-way analysis of variance (Origin_7.0 SR0; Origin lab Corporation, Northampton, MA, United States). Significant effects of treatments were estimated (*p* < 0.05, *p* < 0.01, and *p* < 0.0001).

## Results

### Characterization of Synthetized NPs

**FIGURE 1:**
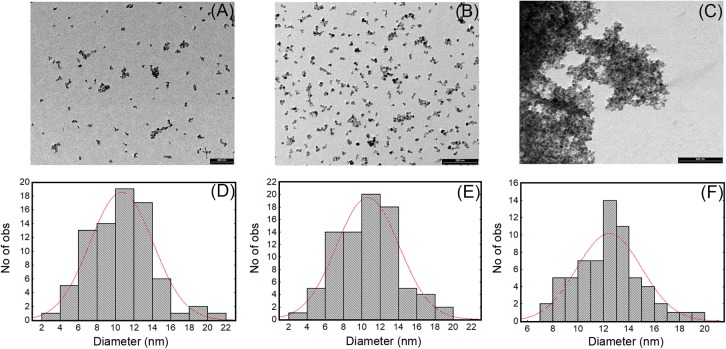
TEM images and size distribution of IONPs **(A,D)**, NP-APTES **(B,E)**, and NP-TEICO **(C,F)**.

**FIGURE 2:**
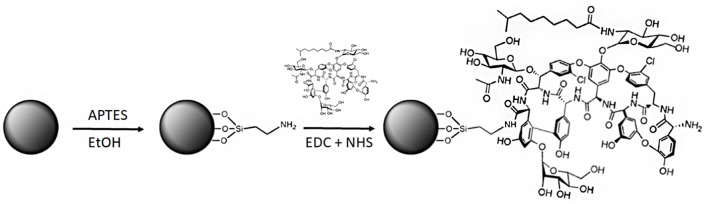
Synthetic route for teicoplanin conjugation to IONPs (not in scale). The first step is functionalization of the IONPs with APTES, followed by the conjugation of teicoplanin by covalent bonding of the terminal carboxylic groups of the antibiotic molecules with the amino groups of NP-APTES via EDC/NHS cross-linking.

Numerous methods for synthesizing IONPs have been reported in the literature (Wu et al., 2015). In this study, we used the coprecipitation method previously optimized by Balzaretti et al. (2017), by which IONPs with good stability and size distribution and no tendency to aggregation could be produced. We confirmed that the NPs obtained had a spherical shape and an average diameter of 10.5 ± 4 nm, as shown by TEM micrograph (**Figures 1A,D**). The functionalization protocol, used to introduce amino groups on the IONPs (**Figure 2**), led to an insignificant increase in the diameter of NP-APTES, which was 10.6 ± 3.6 nm (**Figures 1B,E**). Teicoplanin was conjugated by following a slightly modified protocol, which was previously used for enzyme conjugation (Armenia et al., 2017): carboxylic groups of teicoplanin molecules reacted with the amino groups on the surface of NP-APTES after EDC/NHS antibiotic activation (see below, **Figure 2**). Teicoplanin conjugation led to a more irregular shape of the particles and a slight tendency to aggregation. It is known that correctly conformed teicoplanin molecules tend to dimerize back-to-back in aqueous solutions and that dimerization plays an important role in their biological activity (Treviño et al., 2014). However, this phenomenon was not strong as no NP precipitation occurred. NP-TEICO had an average diameter of 13.6 nm (**Figures 1C,F**).

Transmission electron microscopy observations were complemented by measuring dynamic light scattering (DLS) of the hydrodynamic size of IONPs (**Table 1**); their diameter was estimated to be 14.2 ± 0.5 nm with an average size distribution (PDI) of 0.127, indicating a slight polydispersity typical for the coprecipitation synthesis (Wu et al., 2015). For NP-APTES, an increase in the hydrodynamic diameter (26.8 ± 0.2 nm) due to the presence of the APTES shell around the NP core was registered. The hydrodynamic diameter of NP-TEICO was much larger (568.2 ± 0.6 nm) (**Table 1**), probably due to aggregate formation in the medium used for DLS analysis and to the effect of the glycopeptide side chains and their tendency to dimerize, which might slow down particle diffusion and increase their apparent size (Szpak et al., 2013; **Table 1**). The difference in NP sizes measured by DLS versus TEM is generally attributed to the formation of extra hydrate layers in aqueous solutions (De Palma et al., 2007; Gonçalves et al., 2017). In addition, antibiotic shells conjugated to NPs are usually not sufficiently electron dense to be visible under the electron microscope. The measurement of zeta potential (**Table 1**) showed that the superficial charge of NP-APTES was twofold higher than for IONPs, that is, 22.5 ± 0.2 versus 11 ± 0.8 mV, due to the presence of the amino groups of APTES. A reduction in the surface charge was indeed observed after teicoplanin conjugation: NP-TEICO zeta potential was 12.8 ± 0.6 mV, indicating that NP-APTES were successfully loaded with teicoplanin.

**Table 1:** Physical parameters of synthesized IONPs, NP-APTES, and NP-TEICO.

	Baseline	Polydispersity	Diameter (nm)	Conductance (μS)	Mobility	Z potential (mV)
IONPs	9.9	0.127	14.2 ± 0.5	421	1.5	11.0 ± 0.8
NP-APTES	9	0.18	26.8 ± 0.2	373	1.9	22.5 ± 0.5
NP-TEICO	9	0.189	568.2 ± 0.6	400	1	12.8 ± 0.6


### Preparation of NP-TEICO

Teicoplanin was conjugated to NP-APTES using standard EDC/NHS chemistry: EDC reacted with the carboxylic group of the antibiotic, forming an active O-acylisourea intermediate that could be displaced by the nucleophilic attack of the amino groups present on the NP-APTES surface (**Figure 2**; Hermanson, 2013). Different reaction conditions (reaction medium, EDC/NHS ratio, teicoplanin concentration, time, and temperature of reaction) were explored to improve teicoplanin conjugation on NP-APTES. The quantity of teicoplanin bound to the surface of NP-APTES was estimated by subtracting the unreacted teicoplanin present in the supernatant from the added total antibiotic amount. Teicoplanin was quantified by reverse-phase HPLC as previously reported (Taurino et al., 2011). First trails in water, PBS, and MES buffer indicated that the latter, at pH 6.0, was the most preferable medium for the conjugation reaction (data not shown). As reported in **Table 2**, HPLC analyses confirmed that under the best experimental conditions tested so far, that is, 4 mg/mL of NP-APTES in 30 mM MES buffer, pH 6.0, 13 mM EDC, 26 mM NHS, 500 μg/mL of teicoplanin, the teicoplanin conjugation yield was approximately 90%. Under these conditions, more than 100 μg of teicoplanin was loaded per mg of NP-APTES.

**Table 2:** Reaction conditions tested for teicoplanin conjugation to NP-APTES via EDC/NHS chemistry in 30 mM MES, pH 6.0.

Teicoplanin (μg/mL)	EDC (mM)	NHS (mM)	Temperature (°C)	Time (h)	Yield (%)
100	26	13	4	2	10 ± 0.7
100	26	13	4	4	10 ± 1.5
100	26	13	4	6	12 ± 1.0
100	26	13	25	2	25 ± 0.5
100	26	13	25	4	30 ± 0.4
100	26	13	25	6	65 ± 0.8
100	13	26	25	2	70 ± 1.7
100	13	26	25	4	85 ± 1.4
50	13	26	25	6	100 ± 1.2
100	13	26	25	6	100 ± 0.7
500	13	26	25	6	90 ± 0.9
1000	13	26	25	6	50 ± 0.5


*NP-APTES were used at a concentration of 4 mg/mL. Conjugation yield was calculated by estimating the amount of residual teicoplanin in the reaction medium by HPLC.*

NP-TEICO prepared in this way remained chemically stable when stored at pHs ranging from 5.5 to 7.1 and temperatures from -20 to 25°C. Under these conditions, release of teicoplanin from NP-TEICO was measured by HPLC analysis of incubation buffer; 100% of the antibiotic remained fully attached to NPs for 1 week and decreased by approximately 10% in 1 month (data not shown). Consistently, the antimicrobial activity of NP-TEICO – measured by the antimicrobial susceptibility test versus *S. aureus* ATCC 6538P and *B. subtilis* ATCC 6633 (see below) – was also maintained. After 3 weeks, NP-TEICO maintained from 70 to 90% of its initial antimicrobial activity. Under the same conditions, a water solution of teicoplanin (500 μg/mL) maintained 90% of its initial antimicrobial activity.

### Antimicrobial Activity of NP-TEICO

**FIGURE 3:**
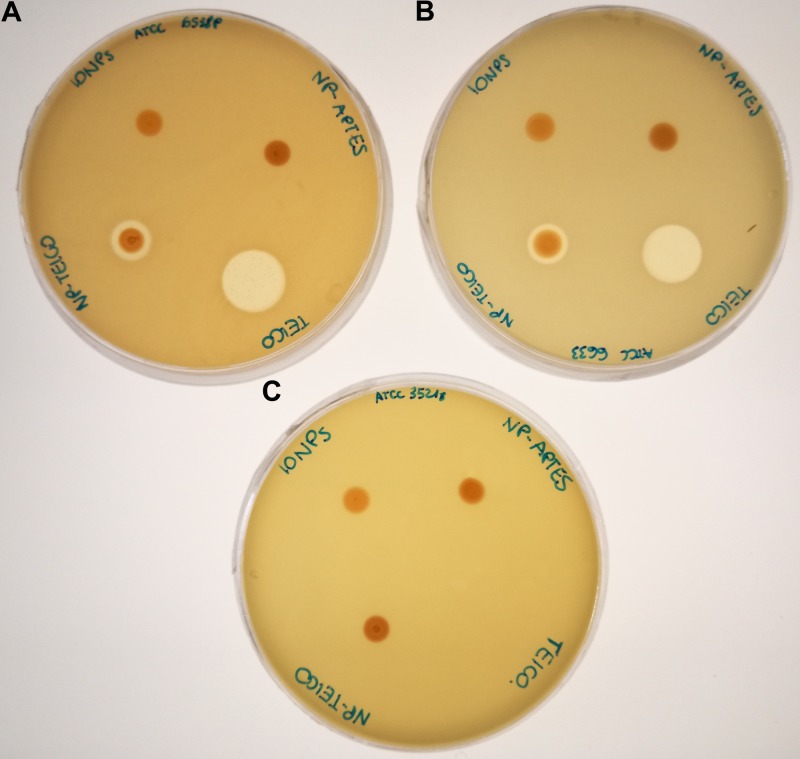
Agar diffusion assay for measuring the antimicrobial activity of IONPs, NP-APTES, NP-TEICO, and non-conjugated teicoplanin versus the two Gram-positive bacteria *S. aureus* ATCC 6538P **(A)** and *B. subtilis* ATCC 6633 **(B)**, and versus the Gram-negative *E. coli* ATCC 35218 **(C)**.

Antibacterial activity of NP-TEICO was initially investigated by comparing the growth inhibitory effects of two commonly used representative species of Gram-positive bacteria, that is, *S. aureus* ATCC 6538P and *B. subtilis* ATCC 6633, and the Gram-negative *E. coli* ATCC 35218, using an agar diffusion assay. **Figure 3** reveals that NP-TEICO inhibited the growth of *S. aureus* and *B. subtilis*, whereas no inhibition halos were observed for *E. coli*, thus demonstrating that NP-TEICO maintained the typical activity and spectrum of action of teicoplanin. Sizes of inhibition halos for the nanoconjugated teicoplanin were not comparable with the ones determined by the non-conjugated teicoplanin, as expected, considering the probably slower diffusion rate of NP-loaded antibiotic in agar medium. Conversely, IONPs and NP-APTES did not show any inhibition halos toward either the Gram-positive or the Gram-negative bacteria. These data indicate that the antimicrobial activity measured by the agar diffusion assay was conferred to NP-TEICO by the conjugation of the antibiotic and that it was not an intrinsic feature of IONPs.

**Table 3** reports the MICs of nanoconjugated and non-conjugated teicoplanin toward clinically relevant strains of *S. aureus* and *E. faecalis*. Although the potency of nanoconjugated teicoplanin was slightly reduced in comparison with the non-conjugated antibiotic, NP-TEICO maintained a valuable antibiotic activity against MRSA and on vancomycin-resistant *E. faecalis* with a VanB phenotype. MICs and MBCs, and consequently the tolerance levels of NP-TEICO toward *B. subtilis*, *S. aureus*, and *E. faecalis*, showed the same trend as those measured for non-conjugated teicoplanin. NP-TEICO and non-conjugated teicoplanin were inactive toward the Gram-negative *E. coli* and toward the vancomycin- and teicoplanin-resistant *E. faecalis* clinical isolate with a VanA phenotype (Van Bambeke, 2006; Binda et al., 2014).

**Table 3:** Comparison of MICs, MBCs, and tolerance levels between non-conjugated and nanoconjugated teicoplanin.

	MIC (μg/mL)		MBC (μg/mL)		Tolerance level	
			
	Non-conjugated teicoplanin	Nanoconjugated teicoplanin	Non-conjugated teicoplanin	Nanoconjugated teicoplanin	Non-conjugated teicoplanin	Nanoconjugated teicoplanin
*B. subtilis* ATCC 6633	2	2	>128	>128	>64	>64
*S. aureus* ATCC 6538P (MSSA)	1	2	128	128	128	64
*S. aureus* ATCC 43300 (MRSA)	0.5	2	64	>128	128	>64
*E. faecalis* ATCC 29212	0.5	1	32	32	64	32
*E. faecalis* ATCC 51299 (VanB)	0.5	2	64	>128	128	>64
*E. faecalis* 9160188401-EF-34 (VanA)	>128	>128	>128	>128	-	-
*E. coli* ATCC 35218	>128	>128	>128	>128	-	-


*The values represent the average of the data from three independent experiments.*

### Effects of NPs on Bacterial Growth Kinetics and Cell Viability

**FIGURE 4:**
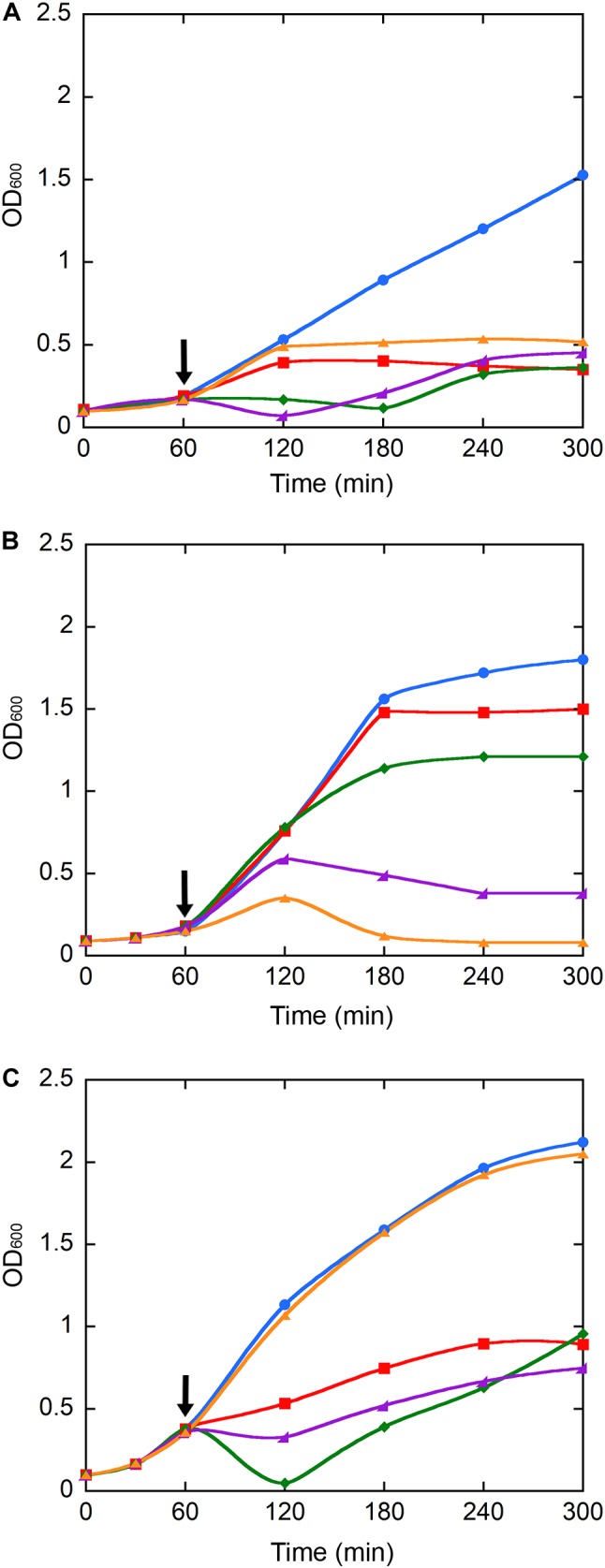
Population growth kinetics of *S. aureus* ATCC 6538P **(A)**, *B. subtilis* ATCC 6633 **(B)**, and *E. coli* ATCC 35218 **(C)** exposed to teicoplanin (orange), IONPs (red), NP-APTES (green), and NP-TEICO (violet). Cultures without any addition (blue) were used as controls. Growth was recorded for 5 h. Black arrows indicate the addition (after 1 h of growth) of NP preparations and of teicoplanin to the bacterial populations. Triplicate experiments were conducted for each condition: standard errors were lower than 5%.

As the antimicrobial activity of IONPs and their derivatives is a matter of intensive debate (Auffan et al., 2008; Chatterjee et al., 2011; Borcherding et al., 2014; Arakha et al., 2015a; Ansari et al., 2017), we further investigated the effects of IONPs, NP-APTES, and NP-TEICO on bacterial cell viability, by adding our NP preparations at the log phase of the growth kinetics of *S. aureus* ATCC 6538P, *B. subtilis* ATCC 6633, and *E. coli* ATCC 35218 populations. Cultures with no added NP or to which only teicoplanin was added were used as negative and positive controls. **Figure 4** indicates that the three bacterial species responded differently to NP interaction. *S. aureus* growth kinetics (**Figure 4A**) were dramatically affected by the addition of NP preparations and, as expected, by the treatment with teicoplanin. Albeit with a slightly different kinetics, cell density appeared equally reduced by two-thirds on 5 h of incubation. Indeed, NP-TEICO and non-conjugated teicoplanin drastically reduced the population growth of *B. subtilis*, whereas the effects of IONPs and NP-APTES were clearly less relevant (**Figure 4B**). Finally, teicoplanin was completely inactive toward the Gram-negative *E. coli*, whereas the addition of IONPs, NP-APTES, and NP-TEICO halved the population growth in a comparable mode (**Figure 4C**).

**FIGURE 5:**
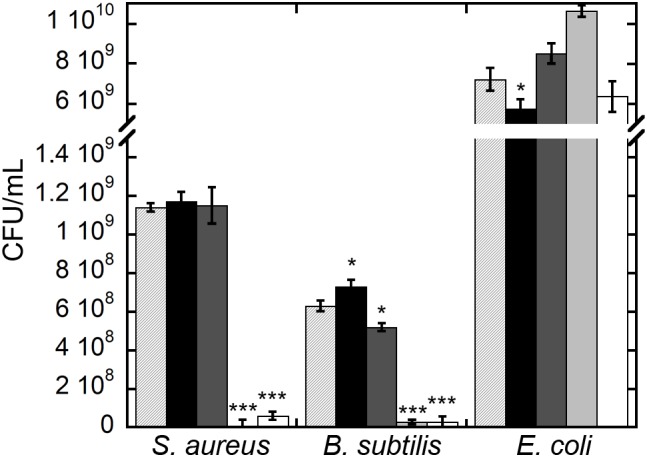
Bacterial cell viability of *S. aureus* ATCC 6538P, *B. subtilis* ATCC 6633, and *E. coli* ATCC 35218 measured as CFUs after 5-h growth (see **Figure 4**) in the presence of IONPs (black bar), NP-APTES (gray bar), NP-TEICO (light gray bar), and teicoplanin (white bar) compared to the untreated control populations (lined bar). Triplicate experiments were conducted for each condition, and the error bars represent the standard errors. One-way ANOVA analyses, ^∗^*p* < 0.05 and ^∗∗∗^*p* < 0.0001.

Significantly, CFU measurements at the end of the growth kinetics reported in **Figure 5** clearly indicate that exposure of Gram-positive bacteria to teicoplanin and NP-TEICO cleared the bacteria population, confirming the comparable antibiotic activity of the nanoconjugated versus the non-conjugated antibiotic (**Figure 5**). As expected, teicoplanin and NP-TEICO were ineffective against *E. coli* cells, which conforms to the antimicrobial spectrum of the antibiotic. In addition, exposure to IONPs and NP-APTES was not bactericidal for any of the tested strains as the cells survived quite well, and in some cases (*E. coli*) even better than the untreated cultures. Thus, we can conclude that NP-TEICO retained an antibiotic activity that was comparable to that of the non-conjugated teicoplanin, whereas IONPs and NP-APTES showed a species-specific transient interaction with bacterial cells, which slowed down population growth but did not kill bacterial cells. This phenomenon merits further investigation.

### Interaction Patterns of NPs With Bacterial Cells

**FIGURE 6:**
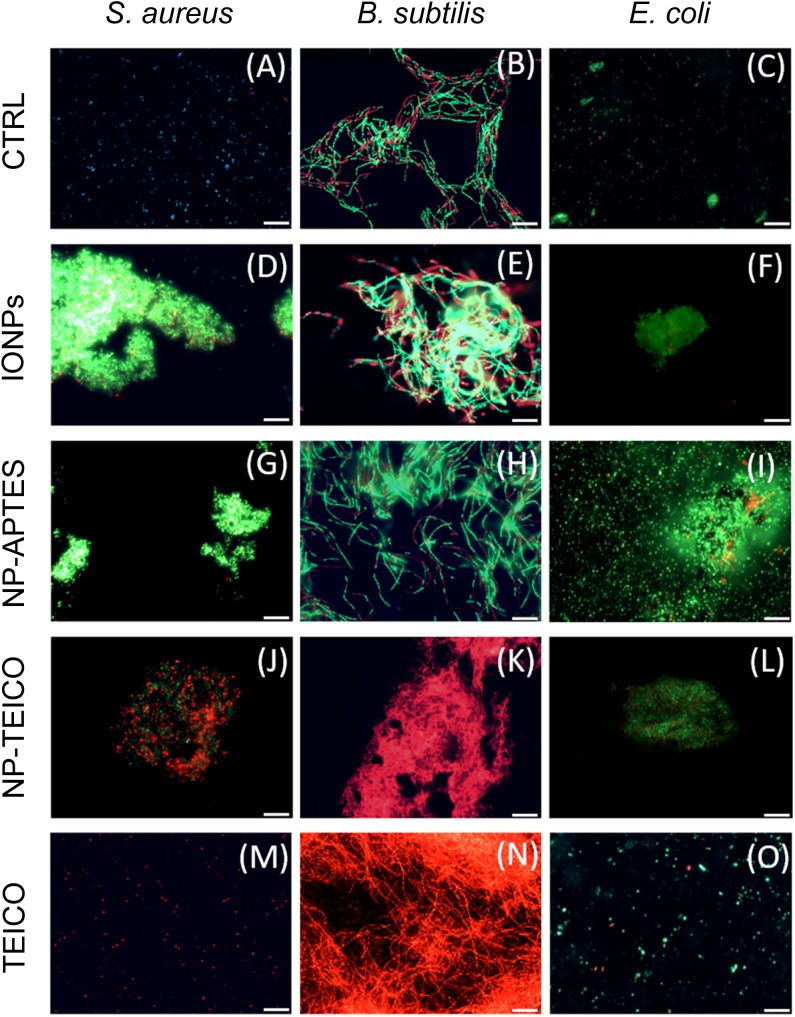
Fluorescence microscopy images of live and dead cells of *S. aureus* ATCC 6538P [first column on the left: **(A,D,G,J,M)**], *B. subtilis* ATCC 6633 [middle column: **(B,E,H,K,N)**], and *E. coli* ATCC 35218 [column on the right: **(C,F,I,L,O)**] in the absence and presence of different NP preparations and of teicoplanin. **(A–C)** untreated cells; **(D–F)** cells treated with IONPs; **(G–I)** cells treated with NP-APTES; **(J–L)** cells treated with NP-TEICO; **(M–O)** cells treated with teicoplanin. Scale bar: 12 μm.

To shed light on the interaction pattern at the IONPs-, NP-APTES- and NP-TEICO-bacteria interfaces, we investigated the effect of adding NP on bacterial cell integrity by using the LIVE/DEAD BacLight fluorescence assay. According to the assay principle and as shown in **Figure 6**, viable cells having an intact cell membrane were stained green by the Syto9 fluorescence dye, whereas non-viable cells with deformed cell membranes were stained red by propidium iodide fluorescence dye (Arakha et al., 2015a). As shown in **Figures 6A–C**, untreated cells of *S. aureus* ATCC 6538P, *B. subtilis* ATCC 6633, and *E. coli* ATCC 35218 exhibited green fluorescence, indicating the presence of 99% viable cells. **Figures 6D–L** show that both Gram-positive and Gram-negative bacteria tended to aggregate on NPs when present. In the presence of IONPs and NP-APTES, the *S. aureus* population exhibited almost 90% of green viable cells (**Figures 6D,G**), whereas more than 50% cells turned to red fluorescence on exposure to NP-TEICO (**Figure 6J**). On the other hand, the *B. subtilis* population exposed to IONPs (**Figures 6E,H**) exhibited the presence of 75% green viable cells, whereas the 95% of *B. subtilis* cells treated with NP-TEICO were red (**Figure 6K**), indicating that nanoconjugated teicoplanin caused a severe loss of membrane integrity and cell viability. Control populations of *S. aureus* and *B. subtilis* treated with non-conjugated teicoplanin exhibited 98% of red non-viable cells (**Figures 6M,N**). In the presence of IONPs, NP-APTES, NP-TEICO, and teicoplanin, the fraction of red fluorescent *E. coli* cells was insignificant compared to untreated cells (**Figures 6F,I,L,O**). Once again, these observations confirm that the three bacterial species responded as expected to nanoconjugated and non-conjugated teicoplanin antibiotic action. They also suggest that naked IONPs and NP-APTES interacted with the different bacteria in a species-specific mode, likely depending on the diverse bacterial surface composition, as already suggested by other authors (Huang et al., 2010; Ebrahiminezhad et al., 2014; Arakha et al., 2015a; Dinali et al., 2017).

**FIGURE 7:**
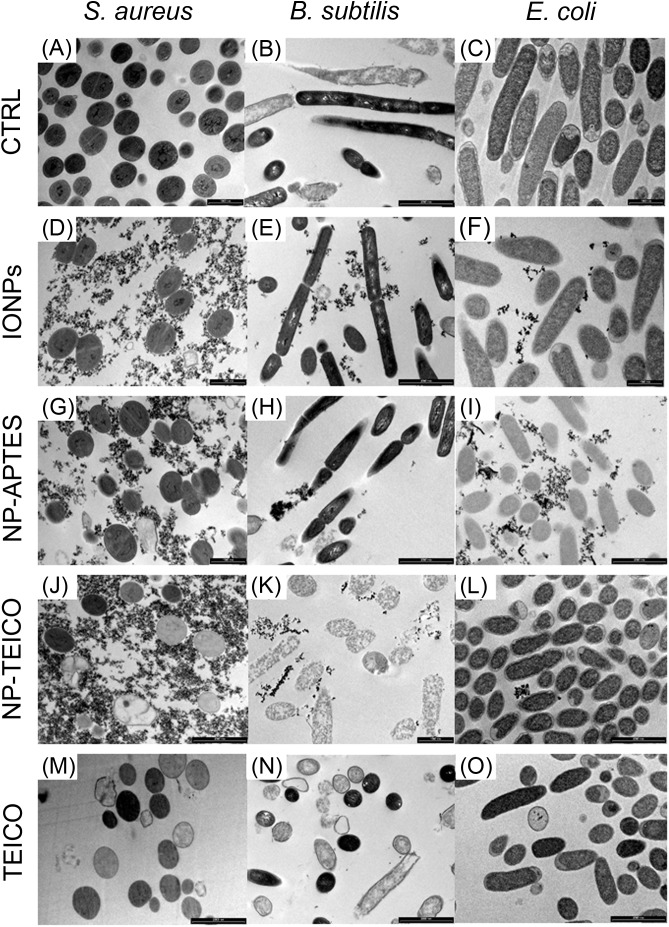
TEM images of *S. aureus* ATCC 6538P [first column on the left: **(A,D,G,J,M)**], *B. subtilis* ATCC 6633 [middle column: **(B,E,H,K,N)**], and *E. coli* ATCC 35218 [column on the right: **(C,F,I,L,O)**] cells in the absence and presence of different NP preparations and of teicoplanin. **(A–C)** untreated cells; **(D–F)** cells treated with IONPs; **(G–I)** cells exposed to NP-APTES; **(J–L)** cells exposed to NP-TEICO; **(M–O)** cells treated with teicoplanin. Scale bars: 1 μm.

**FIGURE 8:**
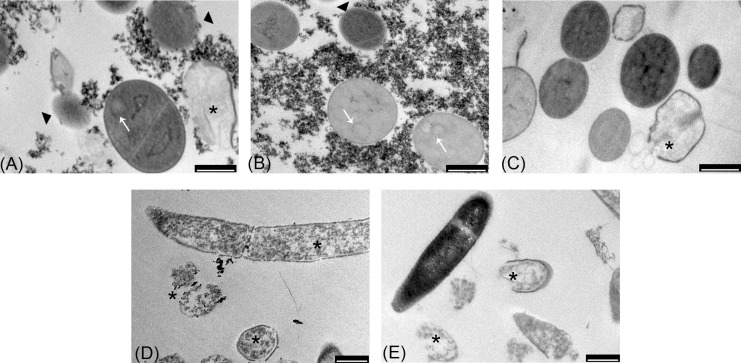
TEM images of *S. aureus* ATCC 6538P **(A–C)** exposed to NP-APTES **(A)**, NP-TEICO **(B)**, teicoplanin **(C)**, and *B. subtilis* ATCC 6633 **(D,E)** exposed to NP-TEICO **(D)** and teicoplanin **(E)**. Scale bar: 500 nm. 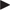
indicates ghost cells; ^∗^indicates lysed cells; white arrows indicate mesosome-like structures.

Transmission electron microscopy images (**Figures 7A,D,G,J,M**) indicated that the exposure of *S. aureus* ATCC 6538P cells to IONPs, NP-APTES, NP-TEICO, and teicoplanin significantly altered cell morphology in comparison to the untreated cells. IONPs, NP-APTES, and, to a significantly greater extent, NP-TEICO interacted with the cell wall of this Gram-positive species. In the presence of NP-APTES, NP-TEICO, and teicoplanin, an increasing percentage of cells without cell walls, so-called ghost cells, became detectable (**Figures 8A,B**). Lysed cells, too, which presented damage in cell walls with cytoplasmic content leaking out, were visible within NP-APTES- and NP-TEICO-treated cells (**Figures 8A,C**). Furthermore, in the presence of NP-APTES and NP-TEICO, intracellular spherical membrane-layered, mesosome-like structures could be detected inside the cells (**Figures 8A,B**). Mesosomes were previously described by other authors (Shimoda et al., 1995; Hartmann et al., 2010), as a consequence of cell membrane damage in *S. aureus* cells treated with antimicrobial peptides such as defensins and gramicidin S.

Conversely, most of the *B. subtilis* ATCC 6633 cells (**Figures 7B,E,H**) exposed to IONPs and NP-APTES showed the same morphology as untreated cells, with undamaged structures, although a few dead or dying cells were detected, characterized by a rough surface and by an interrupted cell membrane. Indeed, the effect of NP-TEICO and teicoplanin on cell integrity was dramatic (**Figures 7K,N**). Cells treated with NP-TEICO and teicoplanin lost their envelope integrity as a consequence of the antibiotic action (**Figures 8D,E**).

No specific alteration in cell morphology was observed in NP- or antibiotic-treated cells of *E. coli* in comparison to the untreated ones (**Figures 7C,F,I,L,O**). Interestingly, in this case, IONPs and, to a much greater extent, NP-APTES tended to stick to the microorganism envelope, whereas the presence of NP-TEICO impeded this interaction. This observation seems to confirm the occurrence of an unspecific electrostatic interaction between positively charged NP-APTES and the negatively charged external cell membrane of this Gram-negative strain, which was previously suggested by other authors (Kell et al., 2008; Huang et al., 2010; Ebrahiminezhad et al., 2014; Arakha et al., 2015a; Dinali et al., 2017).

### Effect of NPs on *S. aureus* Biofilm

**FIGURE 9:**
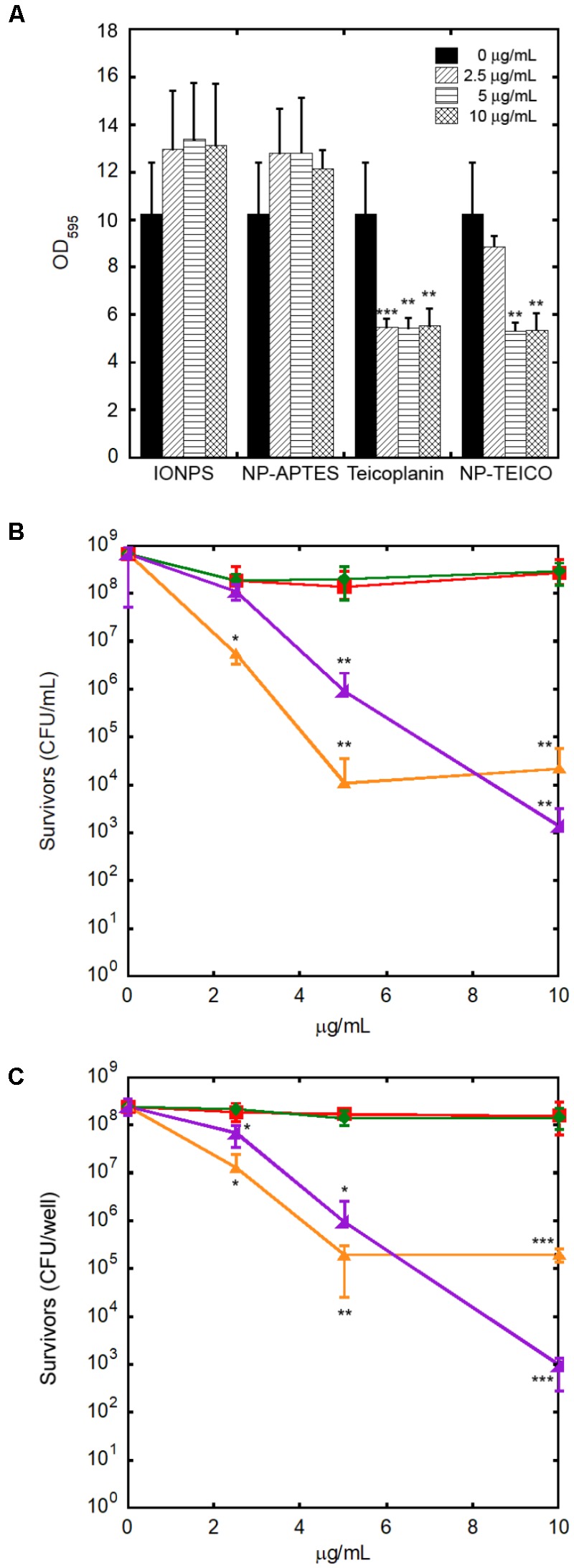
Effect of increasing concentrations of teicoplanin, NP-TEICO, IONPs, and NP-APTES on *S. aureus* ATCC 6538P biofilm formation. In the case of NP preparations, the amounts to be added were defined considering the teicoplanin loaded on IONPs under the conditions defined in the Materials and Methods. Effect on adherent biomass following crystal violet staining **(A)**. Effect on planktonic **(B)** and adherent **(C)** cells exposed to teicoplanin (orange), IONPs (red), NP-APTES (green), and NP-TEICO (violet) on viability assay. The values are expressed as mean ± SD of three independent experiments. One-way ANOVA analyses, ^∗^*p* < 0.05, ^∗∗^*p* < 0.01, and ^∗∗∗^*p* < 0.0001.

Because of the clinical relevance of biofilm infections, the effect of our NP preparations was tested on *S. aureus* ATCC 6538P biofilm formation and eradication. As shown in **Figure 9A**, non-conjugated teicoplanin and nanoconjugated teicoplanin inhibited significantly the biofilm formation at a concentration of 2.5 μg/mL (*p* = 8.03 × 10^-5^) and 5 μg/mL (*p* = 0.002), respectively. No inhibitory effect on biofilm formation was observed after adding IONPs or NP-APTES in comparison to the untreated condition. In the same experimental setting, investigating the effect of IONPs, NP-APTES, NP-TEICO, and teicoplanin on the bacterial viability of adherent and planktonic cell subpopulations gave further information. It was confirmed that IONPs and NP-APTES did not influence the viability of the two subpopulations. Conversely, nanoconjugated and non-conjugated teicoplanin inhibited in a dose-dependent manner the cell viability of both planktonic (**Figure 9B**) and adherent (**Figure 9C**) cells. Teicoplanin at 5 μg/mL caused the decrease of approximately 5 log units in the survival of planktonic cells in comparison to the untreated control cells, whereas the NP-TEICO addition showed a comparable antimicrobial effect at the highest tested concentration of nanoconjugated teicoplanin corresponding to 10 μg/mL (**Figure 9B**). Increasing concentrations of non-conjugated teicoplanin caused a reduction of 2–3 log units in the survival of adherent cells, whereas, notably, the effect of nanoconjugated teicoplanin toward adherent cells was more pronounced (a reduction of 5 log units) than that of non-conjugated teicoplanin at 10 μg/mL and it was statistically significant (*p* = 0.010) (**Figure 9C**). Conversely, neither non-conjugated teicoplanin nor nanoconjugated teicoplanin showed any dispersal effect on 48-h-old biofilms (data not shown), as expected, taking into account that this glycopeptide antibiotic inhibits cell wall synthesis in exponentially growing bacterial cells and is not active on bacterial cells entering into the stationary phase (Binda et al., 2014; Marcone et al., 2018).

### Cytotoxicity of NP-TEICO

**FIGURE 10:**
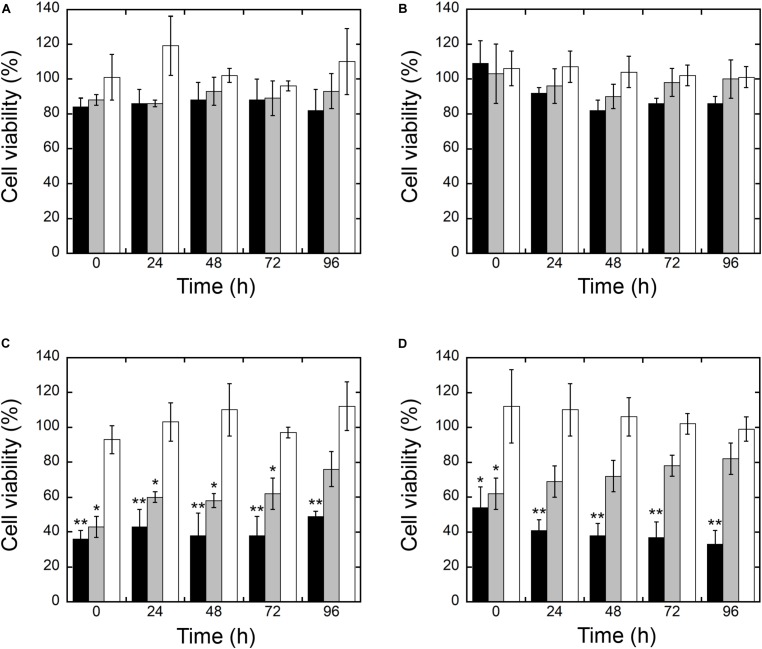
Cell viability of SKOV 3 **(A,C)** and hASC **(B,D)** after different times of exposure to IONPs (black), NP-TEICO (gray), and teicoplanin (white). Cell viability is expressed as a percentage of viable cells compared to the untreated sample, set as 100%. Here, 0.78 μg/mL of non-conjugated or nanoconjugated teicoplanin or 6.24 μg/mL of carrying NPs were added in **(A,B)**; 6 μg/mL of non-conjugated or nanoconjugated teicoplanin or 48 μg/mL of carrying NPs were added in **(C,D)**. In the case of NP preparations, the amounts to be added were defined considering the teicoplanin loaded on IONPs under the conditions defined in the Materials and Methods. The values are expressed as mean ± SD of three independent experiments. One-way ANOVA analyses, ^∗^*p* < 0.05 and ^∗∗^*p* < 0.01.

Cytotoxicity of NP-TEICO was evaluated using two different human cell lines, the well-established immortalized tumor cell line (SKOV-3) (Cappellini et al., 2015) and primary mesenchymal stem cells extracted from human adipose tissue, which are particularly sensitive to nanomaterials (Palombella et al., 2017). Results shown in **Figure 10** indicate that teicoplanin did not exert any effect on the cell viability of either of the human cell lines at any of the tested concentrations. Conversely, both SKOV-3 cells and hASC responded to the exposure of IONPs and NP-TEICO in a concentration-dependent manner. No significant decrease in cell viability was observed after adding nanoconjugated teicoplanin in the range of teicoplanin antibacterial MICs (0.78 μg/mL) (**Figures 10A,B**). The corresponding amounts of carrying NPs did not influence cell viability significantly (**Figures 10A,B**). At a concentration threefold higher than the antibacterial MICs of NP-TEICO (6 μg/mL), the effects of nanoconjugated teicoplanin and of the carrier NPs significantly differed from that of the free antibiotic. Naked IONPs reduced cell viability by more than 60% (after 24 h of exposure) to 50% (after 96 h) in SKOV-3 cells (**Figure 10C**), and by 50% (after 24 h) to 70% (after 96 h) in hASC (**Figure 10D**). NP-TEICO were less cytotoxic, reducing cell viability by 40% (after 24 h) to 20% (after 96 h) in SKOV-3 cells (**Figure 10C**) and by less than 30% (after 24 h) to 20% (after 96 h) in hASC (**Figure 10D**). Interestingly, conjugation of antibiotic molecules to IONPs surface tended to reduce their intrinsic cytotoxicity, as already reported by other authors who demonstrated that covering the NP surface shields toxicity and improves biocompatibility (Javed et al., 2017; Xiang et al., 2017; Xie et al., 2017, 2018).

## Discussion

In the era of antibiotic resistance, the lipoglycopeptide teicoplanin is an extremely important antibiotic used for the prophylaxis and treatment of serious infections caused by Gram-positive bacteria, including MRSA and *E. faecalis* (Van Bambeke, 2006; Marcone et al., 2018). It is used to treat endocarditis, bacteremia, and bone and joint infections. Because of its efficacy and safety, it is used in pediatrics, too. Its spectrum of antibacterial action is similar to that of the previously discovered glycopeptide vancomycin, but teicoplanin has several advantages over vancomycin in the treatment of serious infections: longer half-life, lower nephrotoxicity and ototoxicity, and lack of requirement for serum assays in treated patients. Because of its better stability *in vivo*, it can be administered once a day or with an alternate daily dosage and by intravenous bolus or by intramuscular injection. Oral administration of teicoplanin has also been demonstrated to be effective in the treatment of pseudomembranous colitis and *C. difficile*-associated diarrhea. In addition, teicoplanin is active on some of the vancomycin-resistant enterococci, which are increasingly spreading in hospitals (Binda et al., 2014).

Notwithstanding these important features, to the best of our knowledge, this is the first report of using teicoplanin to functionalize NPs. The teicoplanin molecule has an addressable functional group (the N-terminal carboxylic group of the heptapeptide chain) that we used to covalently bind the amino-activated IONPs. Although there are few published data on optimizing the fabrication of nanoconjugated antibiotics onto IONPs (Lin et al., 2005; Hussein-Al-Ali et al., 2014; Zhu et al., 2015; Dinali et al., 2017), we succeeded in anchoring more than 100 μg of teicoplanin per mg of NP-APTES in this work. The antimicrobial potency of nanoconjugated teicoplanin was slightly lower than that of the non-conjugated counterpart, particularly toward resistant clinical isolates, but NP-TEICO conserved the teicoplanin antimicrobial spectrum of activity toward Gram-positive bacteria and it was particularly active in controlling *S. aureus* biofilm formation. The external membrane of Gram-negative bacteria covering the peptidoglycan layer remained highly impenetrable to both NP-TEICO and teicoplanin, impeding their interaction with the molecular target (Binda et al., 2014). One of the positive features of NP-TEICO prepared in this way was that the formulation maintained chemical stability and antimicrobial activity for at least 1 month. This aspect is relevant, considering that one main advantage of using magnetic antibiotic nanocarriers *in vivo* could be that they can be recovered and recycled after single uses, reducing local dose administration and potential side effects and decreasing the risk of selective pressure on resistant strains. In addition, their targeted delivery to the site of infection/biofilm by using an external magnetic field might increase their *in situ* concentration, potentiating their local efficacy. For this reason, we consider the fact that NP-TEICO inhibited *S. aureus* biofilm formation, conserving the activity of non-conjugated teicoplanin versus the planktonic cells and improving it toward the adherent cells, to be promising. Different non-specific interactions such as electrostatic, hydrophobic, and van der Waal interactions are responsible for adhesion of bacteria on any material surfaces creating biofilms. Thus, it is possible that NP-TEICO anti-biofilm activity is potentiated (in comparison to the non-conjugated antibiotic) by intercepting these non-specific interactions, although in our experiments IONPs and NP-APTES had no effect on biofilm formation.

Unfortunately, *S. aureus* has dramatically re-emerged as a clinically relevant pathogen due to its resistance to antibiotics and the increased use of indwelling clinical devices. Millions of indwelling medical devices are implanted every year, and *S. aureus* is the major culprit for infections and failure of these devices (Arciola et al., 2018). *S. aureus* biofilms are also implicated in chronic wound infections such as diabetic foot ulcers, venous stasis ulcers, and pressure sores, which are quite resistant to antibiotic treatments. Teicoplanin carried by magnetically driven NPs can more easily reach deep tissue infections, which are difficult to treat using topical antibiotics due to the poor tissue penetration, and better penetrate the diffusion barriers that biofilms produce.

In the last decade, a certain level of intrinsic antimicrobial and cytotoxicity activity has been controversially attributed to the IONPs themselves. Although IONPs and NP-APTES have shown some antibacterial effect against diverse Gram-positive and Gram-negative bacteria, the real extent of this phenomenon and the underlying mechanism has hitherto not been well understood (Baranwal et al., 2018). Ansari et al. (2017) reported a dose-dependent antibacterial activity of IONPs against *Bacillus cereus* and *Klebsiella pneumoniae.* In contrast, Auffan et al. (2008) indicated that chemically stable IONPs were not toxic to *E. coli* at 700 mg/L, whereas Chatterjee et al. (2011) reported a dose-dependent effect on *E. coli* cells. Borcherding et al. (2014) showed that IONPs had a positive effect in promoting the growth of *Pseudomonas aeruginosa.* Arakha et al. (2015a) published an illuminating study and demonstrated, by combining a complete set of microbiological and biophysical methods, that IONPs did not show any significant antimicrobial activity toward *B. subtilis* and *E. coli*. Coating IONPs with positively charged chitosan, instead, conferred them with an increased so-called antimicrobial propensity against *B. subtilis* and *E. coli*, which depends on the interfacial interaction between NPs and bacterial surfaces (Arakha et al., 2015a,b).

In the present work, we compared the antimicrobial activity of NP-TEICO with that shown by IONPs and NP-APTES by using a set of methods (agar diffusion assay, BacLight fluorescence assay, bacterial growth kinetics, CFU measurement, and TEM observations) comparable to those previously used by Arakha et al. (2015a,b). Thus, we could conclude that the antibiotic activity of nanoconjugated and non-conjugated teicoplanin differed dramatically from the phenomenon described as antimicrobial propensity, which is based on an electrostatic attraction between cationic NPs and anionic bacterial cell surfaces (Arakha et al., 2015a,b). Electrostatic attraction promotes unspecific adhesion of NPs onto the cell wall of Gram-positive bacteria and the external cell membrane of the Gram-negative bacteria (Qi et al., 2013; Baranwal et al., 2018). This adhesion likely represents the mechanism by which IONPs and, to a greater extent, the positively charged NP-APTES impaired the growth of *S. aureus*, *B. subtilis*, and *E. coli* in our experiments of bacterial growth kinetics. This interfacial effect was transient and reversible, differing from the specific killing activity of teicoplanin and NP-TEICO toward the Gram-positive bacteria. Nevertheless, TEM observations suggested that we cannot completely rule out that cell adhesion of IONPs and of NP-APTES might provoke cytosolic shrinkage and cell membrane detachment (and eventually cell rupture), as observed in *S. aureus* and, with a lower frequency, in *B. subtilis*. In any case, this phenomenon was again sporadic, probably depending on surface composition and on the physiological state of single bacterial cells, as indicated by Dinali et al. (2017).

Although IONPs have been increasingly proposed for a wide range of biomedical applications, such as drug delivery, magnetic resonance imaging, thermal ablation therapy, and treatment of iron-deficient anemia, our understanding of their interaction with animal cells and animal models is still relatively limited (Natan and Banin, 2017; Feng et al., 2018). Recent studies showed that physicochemical properties, including particle size, PDI, surface charge, oxidation state of iron, and different surface coatings, greatly influence their biological effect *in vitro* and *in vivo* (Feng et al., 2018; Wang et al., 2018). Among the super magnetic NPs, IONPs were generally preferred because they are less toxic than those based on nickel and cobalt (Gornati et al., 2016). However, it was recently demonstrated that IONPs can enter eukaryotic cells not only by endocytosis, but also by diffusion through the plasma membrane, gaining direct access to the cytoplasm (Zanella et al., 2017). In addition, the intrinsic catalase-like activity of IONPs might antagonize the accumulation of toxic reactive oxygen species they have induced and thereby modulate the extent of cellular oxidative stress, autophagic activity, and programmed cell death (Wang et al., 2018). In this complex framework, a complete evaluation of the cytocompatibility of our NP-TEICO preparation *in vitro* and *in vivo* systems lies outside the scope of this work, although it would represent a future interesting extension of the study. Here, we demonstrated that at the concentrations that encompass the teicoplanin antibacterial MIC values, teicoplanin coating of IONPs reduced their intrinsic cytotoxicity toward two human cell lines, thus improving their potential biocompatibility. Further intensive *in vitro* and *in vivo* investigations are needed to develop an NP-TEICO-based drug formulation that could be administered systemically or topically to treat deep tissue infections and/or cover medical devices to prevent biofilm formation. Our results indicate that combining synergistically the unique properties of different nanomaterials would represent a good strategy, in this way providing a novel route to prevent and treat bacterial infections and, at the same time, reduce the intrinsic cytotoxicity of NPs, as already indicated by other authors (Xiang et al., 2017; Xie et al., 2017, 2018).

## Author Contributions

IA, GM, GB, and FM conceived the experiments, interpreted the results, and wrote the manuscript. IA developed and produced the NPs and performed the characterization. IA, FB, and GM conducted and interpreted the experiments on the microbiological activity of NPs. VO and EM conducted and analyzed the experiments on biofilms. CP and RG performed the microscopical observations and cell cytotoxicity tests and analyzed the results. All authors reviewed and approved the final manuscript.

## Conflict of Interest Statement

The authors declare that the research was conducted in the absence of any commercial or financial relationships that could be construed as a potential conflict of interest.
